# The Role of Intestinal Microbiota in the Development and Severity of Chemotherapy-Induced Mucositis

**DOI:** 10.1371/journal.ppat.1000879

**Published:** 2010-05-27

**Authors:** Michel J. van Vliet, Hermie J. M. Harmsen, Eveline S. J. M. de Bont, Wim J. E. Tissing

**Affiliations:** 1 Department of Pediatric Oncology/Hematology, Beatrix Children's Hospital, University Medical Center Groningen, University of Groningen, The Netherlands; 2 Department of Medical Microbiology, University Medical Center Groningen, University of Groningen, The Netherlands; University of California San Diego, United States of America

## Abstract

Mucositis, also referred to as mucosal barrier injury, is one of the most debilitating side effects of radiotherapy and chemotherapy treatment. Clinically, mucositis is associated with pain, bacteremia, and malnutrition. Furthermore, mucositis is a frequent reason to postpone chemotherapy treatment, ultimately leading towards a higher mortality in cancer patients. According to the model introduced by Sonis, both inflammation and apoptosis of the mucosal barrier result in its discontinuity, thereby promoting bacterial translocation. According to this five-phase model, the intestinal microbiota plays no role in the pathophysiology of mucositis. However, research has implicated a prominent role for the commensal intestinal microbiota in the development of several inflammatory diseases like inflammatory bowel disease, pouchitis, and radiotherapy-induced diarrhea. Furthermore, chemotherapeutics have a detrimental effect on the intestinal microbial composition (strongly decreasing the numbers of anaerobic bacteria), coinciding in time with the development of chemotherapy-induced mucositis. We hypothesize that the commensal intestinal microbiota might play a pivotal role in chemotherapy-induced mucositis. In this review, we propose and discuss five pathways in the development of mucositis that are potentially influenced by the commensal intestinal microbiota: 1) the inflammatory process and oxidative stress, 2) intestinal permeability, 3) the composition of the mucus layer, 4) the resistance to harmful stimuli and epithelial repair mechanisms, and 5) the activation and release of immune effector molecules. Via these pathways, the commensal intestinal microbiota might influence all phases in the Sonis model of the pathogenesis of mucositis. Further research is needed to show the clinical relevance of restoring dysbiosis, thereby possibly decreasing the degree of intestinal mucositis.

## Introduction

Mucositis, also referred to as mucosal barrier injury, is one of the most debilitating side effects of radiotherapy and chemotherapy treatment [1]. It is characterized by both inflammation and cell loss in the epithelial barrier lining the gastrointestinal tract [2], [3]. Clinically, mucositis is associated with bacteremia, malnutrition, the use of total parenteral nutrition, and an increment in the use of intravenous analgesics. These complications all lead to longer hospitalizations and increasing health care costs. Moreover, mucositis is a frequent reason for reducing the dosages of chemotherapeutics or to postpone chemotherapy treatment, ultimately leading towards a higher mortality in cancer patients [2], [4].

Historically, research has focused on oral mucositis. More recently, attention has been drawn towards the pathophysiology and clinical symptoms of intestinal mucositis, which is characterized by symptoms like nausea, bloating, vomiting, abdominal pain, and severe diarrhea [5], [6].

**Figure 1:**
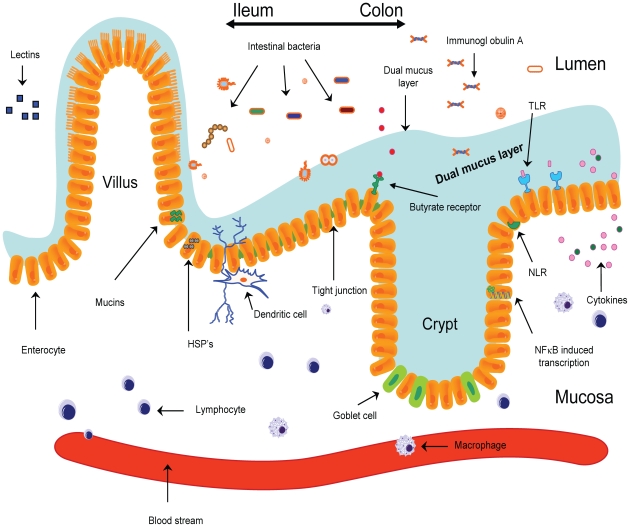
The epithelial barrier is comprised of a single layer of epithelial cells intertwined by tight junctions. The mechanical barrier is increased further by a mucus layer. Binding of bacteria to TLRs present on epithelial cells results in the activation of NFκB, ultimately resulting in the release of pro-inflammatory and anti-inflammatory cytokines. After phagocytosis, bacterial products are internalized and then are recognized by receptors of the NOD family (NLRs), resulting in the modulation of the inflammatory response. Dendritic cells are capable of internalizing bacteria sampled from the lumen, after which bacteria are presented to immune effector cells. HSPs, heat shock proteins; NLR, NOD-like receptor; sIgA, secretory immunoglobulin A; TLR, Toll-like receptor.

**Figure 2:**
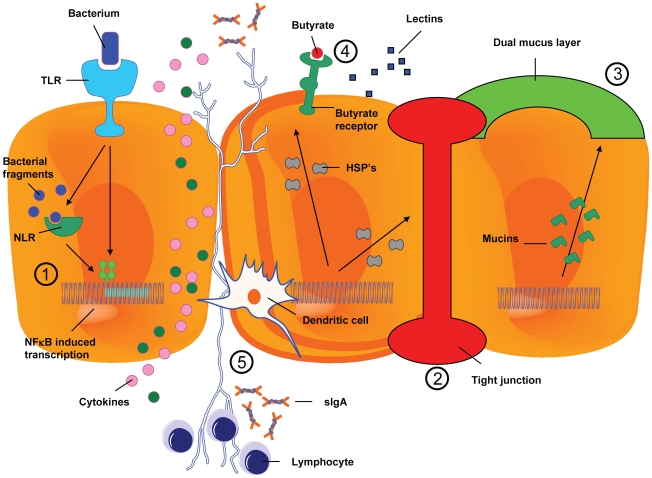
The resident microbiota interferes in the process of mucositis. Depicted are five possible ways in which intestinal bacteria can attenuate or aggrevate mucositis: 1) influencing the inflammatory process, 2) influencing intestinal permeability, 3) influencing the composition of the mucus layer, 4) influencing resistance to harmful stimuli and enhancing epithelial repair, and finally, 5) the activation and release of immune effector molecules.

According to the model introduced by Sonis, five phases are important in the pathophysiology of mucositis: (1) the formation of reactive oxygen species leading to the activation of nuclear factor kappa B (NFκB) during the initiation phase, (2) the induction of messenger molecules such as tumor necrosis factor alpha (TNFα), resulting in treatment-related tissue inflammation and apoptosis during the upregulation/message generation phase, (3) the amplification of messenger molecules in the amplification/signaling phase, leading to more inflammation and apoptosis, (4) discontinuity of the epithelial barrier resulting from apoptosis during the ulcerative phase, thereby promoting bacterial translocation, and (5) a spontaneous healing phase, characterized by cell proliferation [3]. According to this five-phase model, the intestinal microbiota plays no role in the pathophysiology of mucositis. However, research has implicated a role for the commensal intestinal microbiota in several local and systemic inflammatory diseases like inflammatory bowel disease, pouchitis, radiotherapy-induced diarrhea, atopic disease, obesity, and diabetes [7]–[11]. Recent studies have also shown that both chemotherapeutics and (prophylactically used) antibiotics do have an effect on intestinal microbial composition [12]–[14]. Moreover, the effects of the changing commensal intestinal microbiota on the development and severity of mucositis are being unravelled. Research has shown that bacteria play a role in the metabolism of certain chemotherapeutics. The outgrowth of these bacteria might lead to the formation of active toxic metabolites of the chemotherapeutic drug, which directly affects the progression of intestinal mucositis [13]. However, the commensal intestinal microbiota might also have beneficial effects on the development of intestinal mucositis, as the mere presence of resident intestinal bacteria might offer protection against its development. In this review, we propose and discuss five pathways in the development of mucositis that are potentially influenced by the commensal intestinal microbiota: 1) the inflammatory process and oxidative stress, 2) intestinal permeability, 3) the composition of the mucus layer, 4) the resistance towards harmful stimuli and epithelial repair mechanisms, and 5) the activation and release of immune effector molecules (Figures 1 and 2).

## Host–Microbe Interaction

A detailed review of the communication pathways between the intestinal microbiota and the human host is beyond the scope of this article and this communication is therefore only shortly reviewed.

The epithelial barrier lining the gastrointestinal tract is composed of a single layer of epithelial cells intertwined by tight junctions [15]. These epithelial cells have two important functions. Firstly, they form a mechanical barrier separating the inside of the human body from the outside world. Secondly, they are essential in the communication between the human body and the intestinal microbiota [16]–[18].

An important aspect of these two functions of the epithelial cells is the dual mucus layer at the apical side of the epithelial cells [19], [20]. The inner layer strengthens the epithelial barrier, whereas the loose outer layer is proposed to be important in the communication between epithelial cells and microbiota [20], [21].

With respect to the communication between microbes and the gut, two groups of receptors are thought to be important in the communication between the human body and the resident microbiota: the Toll-like receptor (TLR) family and the nucleotide oligomerisation domain (NOD) receptor family [22]–[25]. Both groups of receptors play an important role in the genesis and modulation of the inflammatory response. The TLRs are present at the outer membrane of the epithelial cells. Bacteria are recognized by the extracellularly located part of TLRs, leading to activation of NFκB [23], [25]. In turn, activation of NFκB results in the development of an inflammatory response. So far, multiple members of the TLR family have been described in mammals. The most extensively researched receptors are TLR-2, TLR-3, TLR-4, TLR-5, and TLR-9 [16], [23], [26]–[32]. TLR-2 is activated by peptidoglycan, a part of the cell wall of gram-positive bacteria, whereas TLR-4 is activated by lipopolysaccharide (LPS), a substance of gram-negative microorganisms. TLR-3 is activated by viral DNA, TLR-9 is activated by bacterial DNA, and TLR-5 is activated by the protein flagellin, present in flagellated bacteria. After binding to TLRs, bacteria are processed and bacterial parts are transported intracellularly. Here they bind to receptors of the NOD family. It is believed that activation of NOD receptors modulates the inflammatory response activated by TLR binding [22]. This theory is supported by the fact that NOD−/− mice are profoundly susceptible to intestinal inflammation [33], [34]. Moreover, mutations in NOD2 are associated with the development of Crohn's disease in humans [35]–[37].

Not only epithelial cells, but also local dendritic cells are thought to play a role in the initiation and/or modulation of intestinal inflammation and, in addition, in the induction of tolerance [38]–[40]. Dendritic cells sample bacteria from the intestinal lumen, after which these bacteria are transported to the local lymph nodes. Here, the bacteria are presented to immune cells, whose activation can result in the activation of the innate and adaptive immune system. Why certain microbial stimuli result in tolerance where others induce an inflammatory response is still largely unknown.

## Pathways Describing the Role of Commensal Intestinal Microbiota in Mucositis

### 1) Influencing the Inflammatory Process and Modulating Oxidative Stress

The healthy human intestine is characterized by a state of low-grade inflammation. The resident microbiota guarantees a constant exposure to TLR ligands such as peptidoglycan, LPS, and bacterial DNA. This ensures a continuous basal activation of downstream signaling pathways, resulting in low-grade physiological inflammation [25], [27]. Paradoxically, commensal bacteria are also capable of suppressing more severe inflammatory responses, and their disappearance may even result in incremental inflammation [41]–[46]. For example, *Bacteroides thetaiotaomicron* and *Bifidobacterium infantis* both decrease NFκB activation [43], [47], leading to a decrease in endotoxin levels and plasma interleukin (IL)-6 levels [45]. The *Clostridium* XIVa group has been proposed to attenuate intestinal inflammation by exerting an effect on polyamine secretion, which in turn regulates the expression of TLR-2 [28], [48].

Bacteria or bacterial parts, as well as their secreted products, relieve inflammatory symptoms. For example, *Faecalibacterium prausnitzii* secretes a substance capable of decreasing NFκB activation. This so far unidentified substance induces the production of the anti-inflammatory IL-10, thereby attenuating inflammation. *B. infantis* also secretes an unidentified product that attenuates colitis in mice [46], [49]. Several intestinal bacteria produce short chain fatty acids (SCFAs), with butyrate being the most thoroughly investigated. Butyrate is produced by *F. prausnitzii* and *Clostridium* XIVa and has been shown to have profound anti-inflammatory effects [50]–[54]. Substitution of butyrate attenuates inflammatory symptoms in (diversion) colitis and chemotherapy-induced mucositis in vivo in mice [46], [55]–[58]. Moreover, butyrate not only attenuates inflammation, but also reduces intestinal permeability and stimulates the activation of immune effector molecules.

In short, multiple intestinal bacteria are capable of decreasing NFκB activation, resulting in a diminished production of inflammatory cytokines. The exact nature and relevance of the relationship between chemotherapy-induced mucositis, inflammation, and intestinal microbiota is subject to ongoing research.

### 2) Influencing Intestinal Permeability

Intestinal permeability increases after chemotherapy treatment, and has been shown to be one of the hallmarks of the third and fourth phases of mucositis as reported by Sonis [2], [3], [6]. One of the mechanisms resulting in a chemotherapy-induced increase in permeability is probably villous atrophy. Atrophy leads to an increase of intestinal permeability, as has been shown both in vivo and in vitro [59]. However, the resident intestinal microbiota has also been proposed to influence intestinal permeability [26], [60]. Indeed, several commensal bacteria have been shown to improve the epithelial barrier function both in vitro and in vivo, although not all in vivo studies were able to confirm these improvements [59], [61]–[65]. For example, TLR-2 ligands stimulate the phosphorylation of protein kinase C, leading to a decrease in intestinal permeability [26]. This decrease in permeability is proposed to be the result of changes in tight junctions. Administration of bifidobacteria is associated with an enhanced expression of proteins forming tight junctions [49], and has been shown to decrease intestinal permeability [64]. Both bifidobacteria and lactobacilli have been shown to increase tight junction protein expression and restore intestinal permeability [66]–[68].

Another factor contributing to attenuating intestinal permeability is the bacterial induction of heat shock proteins (HSPs). These HSPs are thought to preserve the viability of epithelial cells in stress conditions [69]–[71], thereby reducing intestinal permeability.

Finally, the bacterial production of SCFAs is associated with a reduction in intestinal permeability. This effect of SCFAs is also proposed to be mediated by an increase in epithelial cell viability [52], [58], [72].

Epithelial cell loss is a hallmark of the third phase of the five-phase mucositis model, eventually resulting in an increased permeability. The commensal intestinal microbiota attenuates cellular atrophy and increases tight junction strength. Therefore, we propose that changes in the commensal intestinal microbiota influence the third phase of mucositis. This way, the commensal intestinal microbiota might influence the eventual severity of mucositis encountered in the ulcerative phase.

### 3) Influencing the Composition of the Mucus Layer

As mentioned before, the mucus layer covering the intestinal epithelium strengthens the mechanical epithelial barrier. The protective mucus layer is comprised of glycoproteins, trefoil factors, and mucins. These mucins are produced by goblet cells, which are specialized epithelial cells [73]. The composition of the mucus layer is important in the protection against bacterial infections and inflammation. For example, it has been shown that mucin type 2 knockout mice develop severe colitis after harmful stimuli, in contrast to mice capable of producing mucin 2. Furthermore, in animals lacking mucin 2, bacteria are detected deep down in the normally sterile crypts of the intestine [20], [74].

The commensal intestinal microbiota is proposed to play a role in the maintenance of the mucus layer. Indeed, the absence of these intestinal microbiota is associated with a decrease in goblet cells, which are also smaller in size [75]. Furthermore, the thickness of the mucus layer is decreased in animals devoid of intestinal microbiota.

The genes encoding mucins are directly regulated by bacteria and their products [76]–[78], and in response to intestinal microbes and/or their secreted products the secretion of mucus increases [76], [79]. For example, both *Lactobacillus rhamnosus* Gorbach and Goldin (GG) and *Lactobacillus plantarum* increase the expression of *MUC-2* and *MUC-3* genes, and *Lactobacillus acidophilus* upregulates *MUC-2* gene expression [77], [80]. Furthermore, bacteria producing butyrate are thought to play a role in the composition of the mucus layer, as butyrate is capable of increasing mucin synthesis as well [52].

The commensal resident microbiota not only interferes with the expression of *MUC* genes, but also interferes with the expression and/or activity of cell glycosyltransferases. These enzymes induce changes in the carbohydrate repertoire of mucins, which might change their efficacy in bacterial defense [81], [82].

Thus, the intestinal microbiota influences the composition of the mucus layer covering the epithelium, thereby increasing the strength of the epithelial barrier. A strengthened barrier decreases the risk of bacterial translocation, thereby possibly attenuating inflammation present in the ulcerative phase of the Sonis mucositis model.

### 4) Influencing Resistance to Harmful Stimuli and Influencing Epithelial Repair

The commensal intestinal microbiota contributes to epithelial repair. In germ-free animals, the mitotic index and cell turnover of epithelial cells are lower as compared to normally colonized animals [83], [84]. Moreover, the transit time of epithelial cells migrating towards the top of the intestinal villi is prolonged [85]. These changes result in a retarded renewal, i.e., a retarded repair, of the intestinal epithelium.

Bacterial induction of NFκB not only controls the physiological state of low-grade inflammation in the intestine, it also stimulates the repair of, for example, mechanical-induced epithelial damage [86]. The importance of bacterial ligands in this process is shown in TLR-4−/− epithelial cells. These cells, which are not capable of recognizing the resident microbiota, exhibit severe repair defects in response to harmful chemical stimuli. This is probably due to a reduced capacity of NFκB-induced cytoprotective factors such as HSPs and IL-6 [25], [29]. When TLR ligands were administered to germ-free mice, this was sufficient to protect them against artificially induced colitis [25].

Bacteria acting as TLR ligands are not the only ones that play an important role in increasing the resistance towards harmful stimuli and enhancing epithelial repair. Again, butyrate plays an important role. Butyrate stimulates the migration of epithelial cells, thereby enhancing mucosal healing [52], [72]. Other bacterial products, such as the peptides secreted by *L. rhamnosus* GG, have been shown to inhibit cytokine-induced apoptosis and promote cell growth, thereby also enhancing mucosal repair [87].

Therefore, we again propose that the commensal intestinal microbiota might attenuate the epithelial damage in the third phase of mucositis. As the commensal intestinal microbiota stimulates epithelial repair mechanisms, it can be hypothesized that the microbiota also attenuates mucositis by influencing the healing phase of mucositis.

### 5) Influencing the Production and Release of Immune Effector Molecules

The commensal intestinal microbiota regulates the expression and release of immune effector molecules. These molecules are pivotal for maintaining intestinal homeostasis [27], [88]–[90]. For example, if the contact between microbiota and intestinal epithelium suddenly increases, the expression of RegIIIγ increases. This C-type lectin has antimicrobial activity and limits bacterial translocation. Furthermore, it maintains intestinal integrity and homeostasis [89], [90].

Another immune effector molecule influenced by the resident microbiota is immunoglobulin A (IgA). IgA is produced by mucosa-associated immune effector cells [81], [90], [91]. Intestinal microbiota is capable of regulating the expression of IgA, which in turn regulates the composition of the intestinal microbiota. For example, suppletion of bifidobacteria is associated with an increase in the expression of secretory IgA [92].

Both live bacteria and their products are capable of upregulating immune effector molecules. For example, SCFAs such as butyrate regulate the production of cathelicidins, which exhibit broad-spectrum anti-bacterial activity against potential pathogens [93].

By influencing the expression and release of immune effector molecules, the commensal intestinal microbiota regulates itself and maintains homeostasis in the intestinal tract. In the end, this will positively influence all five phases described in Sonis's mucositis model.

## Conclusion; an Extended Five-Phase Model for Mucositis

Although the protective role of commensal intestinal bacteria in human disease is increasingly being appreciated, research concerning the relationship between intestinal bacteria and chemotherapy-induced mucositis is still scarce. Most studies that investigate the role of bacteria in human disease have focused on inflammatory bowel disease, which is caused by a chronic inflammatory process instead of the acute damage induced by chemotherapeutics.

In the model introduced by Sonis to explain the pathogenesis of radiotherapy-induced and chemotherapy-induced mucositis, the resident intestinal microbiota played no role [3]. However, recently it has been shown that chemotherapy treatment is associated with a decrease in the number of anaerobic bacteria and a decrease in microbial diversity [14], [94]. Furthermore, the resident intestinal bacteria have been shown to play a role in radiotherapy-induced diarrhea [10]. Moreover, research has shown that a decreasing microbial diversity coincides in time with the development of severe chemotherapy-induced mucositis (M. van Vliet et al., unpublished data). We hypothesize that the commensal intestinal microbiota might play a pivotal role in both radiotherapy-induced and chemotherapy-induced mucositis when the intestine is irradiated or when chemotherapeutics are used that deregulate intestinal microbial homeostasis, as the disappearance of the intestinal microbiota will minimize their protection of enterocytes against harmful stimuli. Further research is needed to show whether the commensal intestinal bacteria should be incorporated as a meaningful factor in Sonis's five-phase model for mucositis. Theoretically, the commensal intestinal microbiota could influence all phases of the pathogenesis of mucositis: the initiation phase, the phase of upregulation and message generation, the phase of amplification and signalling, the ulcerative phase, and the healing phase.

Further research will also have to show the clinical relevance of restoring dysbiosis, thereby possibly decreasing the degree of intestinal mucositis. This would not only increase the quality of life of patients, but could also positively influence treatment intensity, probably decreasing the morbidity and mortality of cancer patients. Completely restoring dysbiosis might be a clinical problem, since whole live bacteria used as probiotics have already been described as causing invasive infections in immunocompromised patients and were associated with increased mortality in patients with severe pancreatitis [95]–[98]. However, it has been shown that substitution of bacterial parts instead of whole live bacteria might be sufficient to attenuate local and systemic inflammation without the risk of invasive infections [30], [99], [100].
